# Dietary Patterns and Their Association with Sociodemographic and Lifestyle Factors in Filipino Adults

**DOI:** 10.3390/nu14040886

**Published:** 2022-02-19

**Authors:** Aileen R. de Juras, Wan-Chen Hsu, Susan C. Hu

**Affiliations:** 1 Department of Public Health, College of Medicine, National Cheng Kung University, Tainan City 701, Taiwan; ardejuras@up.edu.ph (A.R.d.J.); jenny13929@gmail.com (W.-C.H.); 2 Institute of Human Nutrition and Food, College of Human Ecology, University of the Philippines Los Baños, Laguna 4031, Philippines

**Keywords:** dietary patterns, factor analysis, adults, Philippines

## Abstract

Recent studies have investigated dietary patterns to assess the overall dietary habits of specific populations. However, limited epidemiological research has been conducted to explore the unique dietary intakes in low and middle-income countries. This study aims to examine the dietary patterns of Filipino adults and their association with sociodemographic and lifestyle factors. A total of 10,025 adults (≥20 years old) who participated in the 2013 Philippine National Nutrition Survey were included in the analysis. Dietary patterns were derived using factor analysis of 18 food groups from the dietary survey component. Six dietary patterns were identified, namely (1) rice; (2) cereal, milk, sugar, and oil; (3) fruits and miscellaneous food; (4) fish; (5) vegetables and corn; and (6) meat and beverage. Generalized ordered logistic regression analysis indicated that the dietary patterns were associated with different factors, specifically sex, age, educational attainment, marital status, employment status, household size, wealth quintile, smoking status, alcohol consumption, and physical activity. Our findings showed distinct dietary patterns among Filipino adults that were influenced by various sociodemographic and lifestyle parameters. The results of this study have valuable public health implications and the dietary patterns generated can further be used to analyze the link between diet and health outcomes.

## 1. Introduction

Dietary risk is an important driver in most deaths and disabilities due to non-communicable diseases. It pertains to the “aggregated effect of diet quality components consisting of low whole grains, fruits, fiber, legumes, nuts and seeds, omega-3 fatty acids, polyunsaturated fatty acids, vegetables, milk, and calcium; and high sodium, trans fat, red or processed meat, and sugar-sweetened beverages” [1]. Globally, an estimated 7.94 million deaths among adults ≥25 years old were attributable to dietary risks in 2019. It also accounts for more than 10% of disability-adjusted life years in many Asian countries, including the Philippines [1].

Investigating patterns of dietary intake is an alternative approach to determine the complex interaction among food components [2]. Dietary patterns may be generated based on prior knowledge of diets known to be healthy (*a priori* method) or through the application of statistical techniques (*a posteriori* method). The most common methodologies employed in the latter approach are factor analysis and principal component analysis [3,4,5].

Numerous research studies on *a posteriori*-derived dietary patterns of adults have been conducted in Asia [6,7,8,9,10,11,12,13,14,15,16,17,18,19,20,21,22,23,24,25,26,27]. However, the literature mostly focuses on diet–disease relationships. To the best of our knowledge, only one study on food patterns and non-communicable disease risk factors was implemented in the Philippines [6]. Therefore, it is critical to advance research on dietary patterns to improve nutrition programs in low and middle-income countries as well as in specific vulnerable groups. Hence, this study was undertaken to identify the dietary patterns among Filipino adults using a nationwide dataset from the 2013 National Nutrition Survey. We also examined the association between dietary patterns and sociodemographic and lifestyle factors.

## 2. Materials and Methods

### 2.1. Study Population

**Figure 1:**
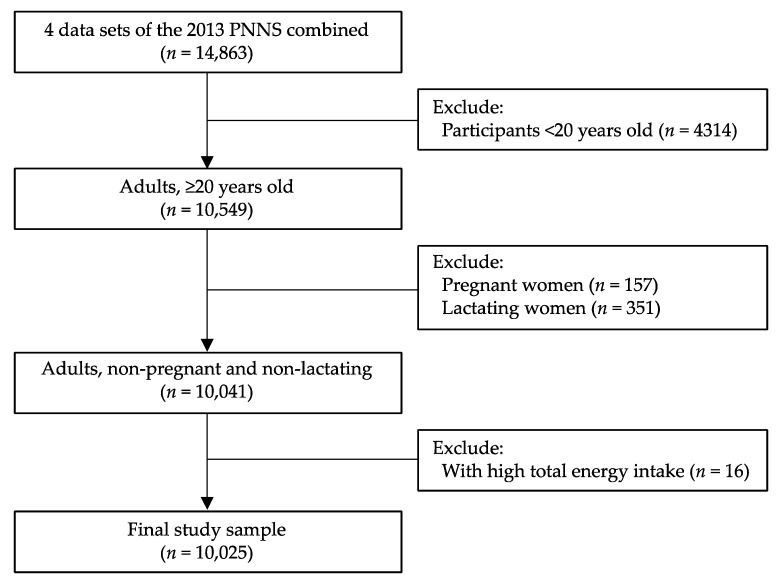
Study participant selection.

We analyzed data from the Philippine National Nutrition Survey (PNNS) carried out by the Food and Nutrition Research Institute, Department of Science and Technology (FNRI-DOST), in 2013 [28]. The PNNS used a stratified multistage sampling design [29,30]. The study samples were selected from participants aged 20 years or older with complete identification data in the four survey components, i.e., dietary, clinical, socioeconomic individual, and socioeconomic household. Pregnant women and lactating mothers were excluded. Participants with high total energy intake or those with greater than 5 SD of the mean energy intake were further excluded, as applied in past studies [31]. A total of 10,025 adults were eligible for the analysis (Figure 1).

### 2.2. Dietary Intake Assessment

Dietary intake was assessed with the 24 h food recall method on two non-consecutive days in the 2013 PNNS. Trained registered nutritionist-dietitians interviewed the participants on their food and beverage consumption from the previous day, starting from the time they woke up until bedtime, using structured questionnaires. The food items recalled were estimated utilizing household measurements or through food sample weighing. Calibrated kitchen utensils (spoons, cups, and plates), rulers, and a photo catalog of foods were shown to the participants as visual aids. Successively, the weights of foods were converted to as purchased values using the list of common foods compiled by the FNRI-DOST. Energy and nutrient intakes were calculated using the Philippine Food Composition Table. The dietary data were aggregated into food groups based on the major functions and state of food [30].

### 2.3. Dietary Pattern Analysis

Dietary patterns were identified using the factor analysis (principal axis factoring procedure in R software) of 18 non-overlapping food groups (Table 1). As suggested in published studies, we excluded the food groups consumed by less than 10% of the population to avoid too many zero values in the data that may lead to extraneous results [32,33,34]. The Bartlett’s test of sphericity (*p* < 0.000) and Kaiser–Meyer–Olkin measure of sampling adequacy (>0.50) were also evaluated prior to factor analysis to verify data suitability. In the identification of the number of factors to be retained, components with an eigenvalue >1.0, the scree plot results, and factor interpretability were considered (Supplementary Table S1 and Supplementary Figure S1). The factors extracted were rotated using varimax rotation to achieve a simpler structure [35]. Food groups with factor loading values of ≥|0.25| were regarded as significantly contributing to a pattern [6,36]. A six-factor solution was finally selected considering to the aforementioned empirical criteria in conjunction with the substantive meaning of the factor loadings.

Factor scores were then estimated for each participant using the regression method. Moreover, because of skewness, the factor scores were divided into tertile intervals to categorize the participant’s adherence to the patterns. The upper tertile (T3) denoted high adherence to a specific dietary pattern and the bottom tertile (T1) denoted low adherence. Labeling of the dietary patterns was carried out according to data interpretation and previous literature. Additionally, a sensitivity analysis was performed to test the stability of the generated factor solution using principal component analysis (PCA). PCA was conducted for the whole sample (dudi.pca function in R software). We compared the number of components to be retained based on eigenvalues, the scree plot analysis, and the variance explained, in which it was consistent (Supplementary Table S2 and Supplementary Figure S2).

### 2.4. Sociodemographic and Lifestyle Factors

The sociodemographic characteristics of the participants included sex (male or female), age (20–39, 40–59, and ≥60 years), education (highest level completed), marital status (single, married/with partner, and others), employment (employed or unemployed), household size (1–3, 4–6, and ≥7), and wealth quintile (poorest, poor, middle, rich, and richest). Household size was created from the socioeconomic datasets [30]. Wealth status was derived using the PCA of household assets, household characteristics, access to utilities, and infrastructure variables. The scores from the analysis were equally divided into five groups to define the wealth quintile as poorest, poor, middle, rich, and richest [30].

The lifestyle factors were comprised of smoking (current smoker or not), alcohol consumption (current drinker or not), and physical activity (low or high). Current smoking was characterized as either: (a) smoking at least one cigarette per day or on a regular/occasional basis, or (b) smoking at least weekly or less often than weekly [30,37]. Current alcohol drinkers referred to the consumption of any alcoholic beverage during the survey period [30,38]. An individual not engaged in (a) 3 or more days of vigorous-intensity activity of at least 20 min daily, or (b) 5 or more days of moderate-intensity activity or walking for at least 30 min per day was classified as having low physical activity [30,37]. All the sociodemographic and lifestyle information were obtained through face-to-face interviews [30].

### 2.5. Statistical Analysis

Descriptive statistics were generated for the sociodemographic and lifestyle characteristics of the participants. Data are presented as weighted percentages and 95% confidence intervals. To assess the association between tertiles of dietary pattern scores and sociodemographic and lifestyle factors, we used the generalized ordered logistic regression analysis. The Brant test was utilized for proportionality assumptions and indicated that the parallel lines assumption was violated for eight variables. Explanatory variables included in the generalized ordered logistic regression model were sex, age, educational attainment, marital status, employment status, household size, wealth quintile, smoking, alcohol consumption, and physical activity. Sampling weights were incorporated in the regression analysis to account for the complex survey sampling design. The significance level was set at *p* < 0.05. All the data analyses were done in R software version 4.0.3 (R Foundation for Statistical Computing, Vienna, Austria).

## 3. Results

### 3.1. Characteristics of Study Participants

The descriptive characteristics of the participants are shown in Table 2. A total of 10,025 adults (4976 men and 5049 non-pregnant and non-lactating women) were included in this study. The sample was predominantly of young adults (47.5%), those who finished high school (37.8%), married individuals (65.5%), employed workers (60.1%), and those who belonged to 4–6 membered families (46.2%). Approximately 23% resided in the richest households or the last wealth quintile. In terms of lifestyle factors, 27.0% were smokers, 51.6% were alcohol drinkers, and 44.7% had low physical activity.

### 3.2. Dietary Patterns and Their Correlates

We identified six dietary patterns through factor analysis (Table 3). Factor 1, named the rice pattern, was characterized by positive loading in the rice and rice products food group, and negative loading in the corn and corn products food group. Factor 2 or the cereal, milk, sugar, and oil pattern was composed of other cereal products, sugar and syrups, milk and milk products, and fats and oils. Factor 3, labeled as the fruits and miscellaneous food pattern, was comprised of fruits and other miscellaneous foods. Factor 4, referred to as the fish pattern, had high loading in the fish and fish products food group. Factor 5 or the vegetables and corn pattern consisted of green leafy and yellow vegetables, other vegetables, and corn and corn products. Factor 6, called the meat and beverage pattern, was constituted of meat and meat products and beverages. Overall, these six dietary patterns explained 21.7% of the variance in the food intake (5.8%, 3.7%, 3.4%, 3.5%, 2.6%, and 2.7% for factors 1–6, respectively).

The results of the generalized ordered logistic regression analysis revealed that the dietary patterns were associated with different sociodemographic and lifestyle factors (Table 4). Males, young adults (20–39 years old), those who were married, those who were living in 4–6 membered households, and adults engaged in high physical activity were more likely to be in the low or medium tertiles of the rice pattern (the first pattern). Furthermore, the odds of adhering to this pattern were found to be significant among individuals with elementary or high school levels of education, unemployed, from the poorest or middle-quintile households, and who were not current smokers. Regarding the cereal, milk, sugar, and oil pattern, which is the second pattern, adults who attained high school and college education or higher, who were employed, who were current alcohol drinkers, and with low physical activity had higher odds of following the pattern. The likelihood of adhering to this pattern increased with the improvement in wealth status. The third dietary pattern or the fruits and miscellaneous food pattern was favored by females, middle-aged (40–59 years old) and older adults (≥60 years old), those who finished college or higher education, and those dwelling in small-sized (1–3 members) households, and the rich or richest-quintile households. Similarly, adults with high school education and non-current smokers had low adherence to this pattern. The odds of adhering to the fourth pattern (the fish pattern) were higher among males and married adults. Males, married individuals, non-current smokers, and adults with high physical activity were more likely to follow the fifth dietary pattern or the vegetables and corn pattern. In addition, this pattern was commonly observed by those belonging to the poorest households, with a significant dose–response relationship. Finally, for the sixth pattern, the meat and beverage pattern, males, adults aged 20–39 years, those with high school and college education attainment, employed workers, those from the richest households or the wealthiest quintile, current smokers, and current alcohol drinkers had a higher likelihood to have low or medium adherence.

In general, females had higher odds of adhering to two dietary patterns (i.e., the cereal, milk, sugar, and oil pattern, and the fruits and miscellaneous food pattern), while the inverse was found in the other dietary patterns. Older adults were more likely to follow the fruits and miscellaneous food pattern. Marital status was significantly associated with three patterns, including the rice, fish, and vegetables and corn patterns. Likewise, the cereal, milk, sugar, and oil dietary pattern, and the meat and beverage dietary pattern were most often observed by current alcohol drinkers. The correlations for wealth status were interesting, as those residing in the poorest quintile had a higher likelihood of adhering to the vegetables and corn pattern, whereas those from the richest quintile favored the cereal, milk, sugar, and oil pattern, and the meat and beverage pattern. The variables of educational attainment, employment status, household size, smoking, and physical activity showed varying associations with different dietary patterns.

## 4. Discussion

This large-scale, population-based study investigated the sociodemographic and lifestyle determinants of the major dietary patterns among Filipino adults. Six distinct dietary patterns were generated, namely (1) rice; (2) cereal, milk, sugar, and oil; (3) fruits and miscellaneous food; (4) fish; (5) vegetables and corn; and (6) meat and beverage. Results of the generalized ordered logistic regression analysis demonstrated that the dietary patterns were differently associated with sex, age, educational attainment, marital status, employment status, household size, wealth quintile, smoking, alcohol consumption, and physical activity.

The dietary patterns derived in our study were in line with previous literature. For example, the rice and fish patterns were comparable to those reported among the Korean [39,40] and Canadian [41] adult populations. The cereal, milk, sugar, and oil pattern contained core food groups similar to the high-energy [26] as well as high-bread and low-rice [17] patterns described in earlier studies. The fruits and miscellaneous food pattern, and vegetables and corn pattern have also been noted in other research [17,42]. Remarkably, the meat and beverage pattern was consistent with the Philippines study [6].

We found significant sociodemographic and lifestyle differences across the six dietary patterns. Men were more likely to adhere to the rice pattern, fish pattern, vegetables and corn pattern, and meat and beverage pattern. This result is in agreement with the national dietary survey results wherein Filipino adult men had a higher mean consumption of rice and rice products, meat, fish, and beverages than women [30]. The influences of age, educational attainment, and marital status on dietary patterns were diverse. Older adults and those with a high school level of education or higher commonly followed the fruits and miscellaneous food pattern, while younger adults and those with at least an elementary education favored the rice pattern; cereal, milk, sugar, and oil pattern; and meat and beverage pattern. In accordance with the literature, age was positively associated with the fruit-based patterns [21,43,44] and was negatively correlated with diets consisting of energy-dense, refined, and processed-food components [21,45]. Our results demonstrated that older adults make positive decisions with respect to their nutrition and health. As for the level of education, most studies documented that healthier dietary patterns or diets containing fruits and vegetables were observed more frequently in highly educated individuals [21,46,47]. Nevertheless, our findings indicate that educational attainment was related to the rice, cereal, milk, sugar and oil; fish; and meat and beverage patterns. This may be ascribed to other factors of healthy eating apart from the number of years in school [48]. With regard to marital status, the relationship found in this study concurs with earlier works wherein dietary patterns may be affected by the state of social relationships [41,49].

The determinants of socioeconomic status, including employment, household size, and wealth quintile, had mixed effects on the dietary patterns. Employed adults had higher adherence to the cereal, milk, sugar, and oil pattern, and the meat and beverage pattern, but had a lower likelihood of following the rice pattern. Moreover, those who lived in small and medium-sized households usually followed the fruits and miscellaneous food pattern, and rice pattern, respectively. The odds of adhering to the cereal, milk, sugar, and oil, and the meat and beverage patterns were 3 to 5 times higher among adults in the richest quintile than the poorest quintile. Socioeconomic position could modify dietary patterns through economic capacity. According to FNRI-DOST, the daily per capita food cost of a typical Filipino household was estimated at PhP 60.39 (USD 1.20) in 2013, with nearly 38% spent on fish, meat, and poultry. The unexpended amount was allocated for cereal and cereal products (30.5%); vegetables (8.3%); miscellaneous food (6.7%); milk and milk products (4.7%); eggs (2.7%); fruits (2.6%); fats and oils (2.4%); sugars and syrup (2.1%); dried beans, nuts, and seeds (1.1%); and starchy roots and tubers (1.0%). In addition, family size altered diet diversity as more food groups were eaten by households with less members. An increasing intake of less expensive food items was also reported among the poorest households [30].

Adults who were current alcohol drinkers and with low physical activity were more likely to adhere to the cereal, milk, sugar, and oil pattern, and those who were both current smokers and alcohol drinkers mostly observed the meat and beverage pattern. Conversely, non-current smokers and those engaged in high physical activity favored the vegetables and corn pattern and rice pattern. Existing evidence relates the clustering of unhealthy diets with unhealthy behaviors such as smoking, alcohol consumption, and physical inactivity [45,50].

A number of limitations should be taken into account in the interpretation of our study results. First, dietary intake was assessed using two non-consecutive 24 h food recalls. Measurement errors and recall bias are inevitable in this method. In order to standardize data collection and minimize errors, the nutritionist-dietitians received trainings before the conducting of PNNS [30]. Furthermore, Denova-Gutiérrez and colleagues [51] conveyed a reasonable validity between the 24 h food recall and food frequency questionnaire in generating dietary patterns using factor analysis, rationalizing the usefulness of food recall. The variability of seasonal food intake was also captured since the survey was carried out for over a year [30]. Second, the factor analysis approach involved several subjective decisions that could influence the constitution of dietary patterns [2,3,4,52]. Nonetheless, this statistical method provides an estimate of the relationship between the food groups consumed by individuals and allows for the determination of dietary patterns that represent the eating habits of a study population [2,52,53,54]. It is encouraging that dietary patterns in our current study were also ascertained in other investigations, which could imply reproducibility among different populations. Third, the dietary patterns explained the low variability of the total food intake (ranging from 2.6 to 5.8%). Past literature elucidates that the amount of variance explained by factors or components determined through *a posteriori* techniques is relatively low and affected by the number of food groups incorporated in the analysis [55,56]. Lastly, the cross-sectional design did not allow for the exploration of lifetime dietary intake and inference of causal relationships. It will be necessary to conduct prospective studies to verify our findings.

Notwithstanding the aforesaid limitations, this study contributes to the current body of knowledge on food patterns mainly because the data was from a national survey that is representative of the consumption behavior of Filipino adults. Another important strength is the sensitivity analysis performed to evaluate the stability of the derived dietary patterns. The results of the factor solution cross-validation through PCA revealed adequate stability.

## 5. Conclusions

This study offers a novel approach in characterizing the consumption patterns of a nationally representative sample of adults in the Philippines. Findings indicate six major dietary patterns among community-dwelling adults, including (1) rice; (2) cereal, milk, sugar, and oil; (3) fruits and miscellaneous food; (4) fish; (5) vegetables and corn; and (6) meat and beverage patterns. These patterns were associated with sociodemographic and lifestyle factors, and would have valuable implications for public health interventions. For instance, it is crucial to tailor nutrition and health promotion programs for adults who are younger, employed, from the richest households, and with unhealthy lifestyles to include fish, fruits, and vegetables in their daily diets. This study also puts forward the concept that healthy dietary patterns in conjunction with food-based dietary guidelines are necessary for improving the nutritional state of adults. Future research is warranted to examine the link between the Filipino diet and the health outcomes using these dietary patterns.

## Figures and Tables

**Table 1:** Food groups used in the dietary pattern analysis ^1^.

Food Groups	Food Items Included
Rice and rice products	Rice and other rice products, such as rice noodles and rice cakes
Corn and corn products	Milled corn, corn on a cob, and other corn products like cornstarch, corn pudding, popcorn, and corn chips
Other cereal products	*Pandesal*, bread, cookies/biscuits, cakes/pastries, noodles, flour, and others
Starchy roots and tubers	Sweet potatoes and products, potatoes and products, cassava and products, and other roots and tubers such as yam, taro, and arrowroot
Sugar and syrups	Sugars, jams, candies, honey, sweetened soda, sherbet, ice drop, ice candy, sugary foods like chocolates, and others
Dried beans, nuts, and seeds	Mungbean and products, soybeans and products, nuts and products, and other dried beans/seeds and products like almond, peas, sesame seed, green peas, tofu, and others
Green leafy and yellow vegetables	Green leafy vegetables, squash fruit, carrot, and other yellow vegetables
Other vegetables	Eggplant, string beans, bitter gourd, other wild vegetables, and other canned/processed vegetables
Fruits	Mango, citrus fruits, strawberry, guava, banana, watermelon, melon, jackfruit, pineapple, young coconut, and others
Fish and fish products	Fresh fish, dried fish, processed fish, crustaceans, and mollusks
Meat and meat products	Fresh meat, organ meat, and processed meat
Poultry	Chicken and other fowls like duck, goose, pigeon, turkey
Eggs	Hen’s egg, duck’s egg, and other eggs like quail egg and turkey egg
Milk and milk products	Fresh whole milk, evaporated milk, recombined milk, powdered milk, condensed milk, cheese, and other milk products like ice cream, yogurt, and cultured milk
Fats and oils	Cooking oil, coconut meat, coconut cream, pork drippings and lard, butter, margarine, peanut butter, and others
Beverages	Coffee, tea, alcoholic beverages, chocolate-based beverages, fruit-flavored drink, and others
Condiments and spices	Salt, vinegar, catsup, and other seasonings
Other miscellaneous food	Lemongrass, bay leaves, oregano, turmeric, food coloring, and others

^1^ The food groups and food items were pre-identified in the 2013 Philippine National Nutrition Survey.

**Table 2:** Characteristics of the participants by sex (*n* = 10,025).

Variables ^1^	%	95% CI
Sex		
Male	49.5	48.3, 50.6
Female	50.5	49.4, 51.7
Age group		
20–39 years	47.5	46.0, 49.0
40–59 years	37.6	36.3, 39.0
≥60 years	14.9	13.9, 15.9
Educational attainment		
Elementary and lower	30.8	28.6, 33.0
High school	37.8	36.1, 39.6
College and higher	31.4	29.2, 33.6
Marital status		
Single	24.5	22.9, 26.1
Married	65.5	63.8, 67.2
Others	10.0	9.2, 10.9
Employment status		
Employed	60.1	58.6, 61.5
Unemployed	39.9	38.5, 41.4
Household size		
1–3	31.5	29.3, 33.9
4–6	46.2	43.4, 49.1
≥7	22.2	19.8, 24.8
Wealth quintile		
Poorest	17.1	14.9, 19.7
Poor	18.6	16.6, 20.7
Middle	20.5	18.5, 22.7
Rich	20.7	18.7, 22.9
Richest	23.1	20.5, 26.0
Current smoker		
Yes	27.0	25.5, 28.5
No	73.0	71.5, 74.5
Current alcohol drinker		
Yes	51.6	49.6, 53.7
No	48.4	46.3, 50.4
Physical activity		
Low	44.7	42.0, 47.3
High	55.3	52.7, 58.0

Table displays weighted percentages. ^1^ Variables with missing observations: educational attainment (*n* = 53), smoking and drinking status (*n* = 581), and physical activity classification (*n* = 701).

**Table 3:** Factor loadings for the six identified dietary patterns.

Food Groups	Dietary Patterns ^1^					
	Rice	Cereal, Milk, Sugar, and Oil	Fruits and Miscellaneous Food	Fish	Vegetables and Corn	Meat and Beverage
Rice and rice products	**0.936**	−0.009	−0.001	0.142	0.064	0.069
Corn and corn products	**−0.331**	−0.099	−0.045	0.046	**0.294**	0.004
Other cereal products	−0.024	**0.475**	−0.005	−0.040	−0.071	0.092
Starchy roots and tubers	−0.046	−0.003	0.037	0.007	0.145	0.017
Sugar and syrups	0.049	**0.327**	0.029	0.004	−0.011	0.198
Dried beans, nuts, and seeds	0.051	0.081	−0.027	−0.070	0.049	0.023
Green leafy and yellow vegetables	0.007	−0.105	0.003	−0.002	**0.491**	−0.073
Other vegetables	0.119	0.038	0.042	−0.118	**0.295**	−0.049
Fruits	−0.007	0.077	**0.570**	0.023	0.065	0.000
Fish and fish products	0.113	−0.031	0.018	**0.741**	−0.054	−0.050
Meat and meat products	0.072	0.205	0.016	−0.133	−0.073	**0.525**
Poultry	0.077	0.208	0.028	−0.079	−0.038	0.153
Eggs	0.103	0.185	0.026	−0.088	−0.042	0.016
Milk and milk products	−0.055	**0.281**	0.089	−0.008	−0.003	0.057
Fats and oils	0.028	**0.266**	0.024	0.024	0.005	0.003
Beverages	−0.009	0.054	−0.008	0.018	0.004	**0.312**
Condiments and spices	−0.039	0.173	−0.007	0.109	−0.024	0.145
Other miscellaneous	0.030	0.058	**0.514**	−0.001	0.050	0.004
Proportion variance, %	5.8	3.7	3.4	3.5	2.6	2.7
Cumulative variance, %	5.8	9.5	12.9	16.4	19.0	21.7

^1^ Dietary patterns are labeled based on the factor loadings with the absolute value of 0.25 or greater. Bold values represent food groups kept in their related dietary pattern.

**Table 4:** Sociodemographic and lifestyle factors associated with dietary patterns analyzed by generalized ordered logistic regression ^1^.

Variables	Rice Pattern		Cereal, Milk, Sugar, and Oil Pattern		Fruits and Miscellaneous Food Pattern		Fish Pattern		Vegetables and Corn Pattern		Meat and Beverage Pattern	
	T2 and T3 vs. T1	T3 vs. T1 or T2	T2 and T3 vs. T1	T3 vs. T1 or T2	T2 and T3 vs. T1	T3 vs. T1 or T2	T2 and T3 vs. T1	T3 vs. T1 or T2	T2 and T3 vs. T1	T3 vs. T1 or T2	T2 and T3 vs. T1	T3 vs. T1 or T2
Sex (ref. = male)												
Female	**0.18** **(0.16, 0.21)**	**0.29** **(0.25, 0.33)**	0.96 (0.85, 1.09)	**1.18** **(1.05, 1.33)**	**1.35** **(1.19, 1.53)**	**1.83** **(1.59, 2.11)**	**0.64** **(0.57, 0.72)**	**0.76** **(0.67, 0.86)**	**0.63** **(0.56, 0.70)**	**0. 60** **(0.53, 0.67)**	**0.73** **(0.64, 0.82)**	**0.82** **(0.72, 0.93)**
Age group (ref. = 20–39 years)												
40–59 years	**0.75** **(0.67, 0.85)**	**0.80** **(0.71, 0.92)**	**0.84** **(0.74, 0.96)**	**0.87** **(0.76, 1.00)**	**1.20** **(1.07, 1.36)**	**1.15** **(1.02, 1.31)**	1.04 (0.93, 1.17)	1.10 (0.97, 1.24)	**1.20** **(1.06, 1.36)**	1.07 (0.95, 1.20)	**0.75** **(0.66, 0.86)**	**0.78** **(0.68, 0.89)**
≥60 years	**0.36** **(0.30, 0.43)**	**0.45** **(0.38, 0.54)**	**0.81** **(0.67, 0.97)**	0.87 (0.72, 1.04)	**1.65** **(1.40, 1.94)**	**1.88** **(1.58, 2.25)**	1.04 (0.88, 1.22)	1.16 (0.97, 1.39)	1.05 (0.88, 1.26)	0.95 (0.80, 1.13)	**0.51** **(0.42, 0.61)**	**0.58** **(0.48, 0.70)**
Educational attainment (ref. = ≤ elementary)												
High school	1.07 (0.94, 1.21)	**1.29** **(1.12, 1.48)**	**1.77** **(1.51, 2.07)**	**1.73** **(1.52, 1.97)**	**1.29** **(1.13, 1.47)**	1.03 (0.89, 1.20)	0.90 (0.79, 1.03)	0.91 (0.79, 1.05)	0.94 (0.82, 1.07)	0.97 (0.85, 1.10)	**1.47** **(1.28, 1.68)**	**1.28** **(1.13, 1.46)**
≥College	**0.83** **(0.70, 0.97)**	0.93 (0.76, 1.13)	**2.52** **(2.05, 3.09)**	**2.37** **(1.94, 2.90)**	**1.58** **(1.33, 1.89)**	**1.22** **(1.04, 1.44)**	0.92 (0.77, 1.09)	**0.85** **(0.72, 0.99)**	0.95 (0.81, 1.12)	0.89 (0.74, 1.08)	**2.01** **(1.70, 2.38)**	**1.69** **(1.42, 2.02)**
Marital status (ref. = single)												
Married	**1.21** **(1.05, 1.38)**	**1.23** **(1.06, 1.43)**	0.95 (0.82, 1.09)	0.92 (0.79, 1.07)	1.08 (0.94, 1.23)	1.01 (0.88, 1.17)	**1.30** **(1.13, 1.49)**	**1.36** **(1.18, 1.57)**	**1.19** **(1.03, 1.38)**	**1.19** **(1.04, 1.35)**	0.89 (0.77, 1.03)	0.96 (0.81, 1.13)
Others	0.84 (0.65, 1.07)	0.88 (0.72, 1.09)	1.03 (0.83, 1.28)	1.02 (0.82, 1.26)	0.90 (0.73, 1.10)	0.93 (0.75, 1.15)	1.07 (0.87, 1.33)	1.03 (0.84, 1.26)	0.89 (0.71, 1.11)	0.82 (0.65, 1.03)	0.86 (0.68, 1.08)	0.90 (0.73, 1.12)
Employment status (ref. = employed)												
Unemployed	**1.15** **(1.02, 1.29)**	1.08 (0.97, 1.20)	**0.87** **(0.78, 0.97)**	**0.81** **(0.72, 0.92)**	0.94 (0.83, 1.05)	1.08 (0.95, 1.24)	0.95 (0.85, 1.06)	1.02 (0.91, 1.13)	0.98 (0.88, 1.10)	0.90 (0.80, 1.00)	**0.71** **(0.63, 0.80)**	**0.74** **(0.67, 0.83)**
Household size (ref. = 1–3)												
4–6	**1.14** **(1.01, 1.30)**	**1.18** **(1.04, 1.36)**	0.92 (0.80, 1.06)	0.96 (0.83, 1.11)	**0.86** **(0.75, 0.98)**	**0.87** **(0.77, 0.99)**	1.09 (0.95, 1.25)	1.03 (0.90, 1.18)	0.98 (0.86, 1.12)	0.98 (0.86, 1.13)	0.98 (0.84, 1.14)	0.92 (0.80, 1.06)
≥7	0.96 (0.81, 1.14)	0.99 (0.83, 1.19)	0.99 (0.82, 1.20)	0.92 (0.77, 1.10)	0.85 (0.71, 1.01)	0.92 (0.78, 1.09)	0.96 (0.80, 1.14)	0.93 (0.78, 1.11)	1.00 (0.84, 1.21)	1.00 (0.84, 1.20)	0.92 (0.77, 1.11)	1.02 (0.85, 1.21)
Wealth quintile (ref. = poorest)												
Poor	0.98 (0.82, 1.18)	1.24 (0.99, 1.54)	1.26 (1.00, 1.59)	**1.47** **(1.24, 1.75)**	1.09 (0.89, 1.32)	1.05 (0.88, 1.26)	0.99 (0.81, 1.21)	1.03 (0.84, 1.27)	**0.77** **(0.64, 0.94)**	0.90 (0.73, 1.12)	**1.39** **(1.12, 1.72)**	**1.22** **(1.01, 1.46)**
Middle	1.06 (0.87, 1.29)	**1.40** **(1.12, 1.77)**	**1.84** **(1.44, 2.34)**	**2.48** **(2.06, 3.00)**	1.21 (0.98, 1.50)	1.16 (0.96, 1.39)	0.99 (0.81, 1.21)	1.02 (0.83, 1.26)	**0.53** **(0.43, 0.66)**	**0.76** **(0.61, 0.94)**	**1.72** **(1.37, 2.16)**	**1.62** **(1.32, 1.99)**
Rich	0.88 (0.72, 1.08)	1.18 (0.93, 1.49)	**2.68** **(2.08, 3.45)**	**3.66** **(2.92, 4.59)**	**1.68** **(1.37, 2.05)**	**1.55** **(1.27, 1.89)**	0.94 (0.75, 1.17)	0.92 (0.75, 1.13)	**0.45** **(0.37, 0.56)**	**0.71** **(0.57, 0.90)**	**3.30** **(2.61, 4.18)**	**3.19** **(2.57, 3.96)**
Richest	**0.62** **(0.49, 0.79)**	0.78 (0.60, 1.03)	**3.71** **(2.83, 4.85)**	**5.63** **(4.35, 7.30)**	**2.21** **(1.78, 2.74)**	**1.87** **(1.50, 2.33)**	0.90 (0.71, 1.15)	0.84 (0.67, 1.06)	**0.40** **(0.32, 0.50)**	**0.58** **(0.44, 0.75)**	**4.41** **(3.43, 5.68)**	**3.79** **(2.95, 4.87)**
Current smoker (ref. = yes)												
No	**1.17** **(1.02, 1.33)**	0.95 (0.82, 1.09)	1.05 (0.91, 1.20)	0.96 (0.83, 1.11)	**1.25** **(1.09, 1.42)**	0.99 (0.87, 1.11)	1.09 (0.95, 1.25)	1.10 (0.95, 1.28)	**1.21** **(1.07, 1.36)**	**1.19** **(1.04, 1.37)**	**0.83** **(0.72, 0.96)**	**0.80** **(0.70, 0.92)**
Current alcohol drinker (ref. = yes)												
No	0.90 (0.80, 1.02)	0.93 (0.82, 1.05)	**0.83** **(0.73, 0.93)**	**0.86** **(0.76, 0.97)**	1.06 (0.94, 1.18)	1.06 (0.94, 1.20)	0.93 (0.82, 1.05)	0.95 (0.83, 1.08)	0.92 (0.81, 1.04)	1.06 (0.93, 1.19)	**0.66** **(0.58, 0.75)**	**0.74** **(0.65, 0.83)**
Physical activity (ref. = high)												
Low	**0.81** **(0.72, 0.92)**	**0.87** **(0.77, 0.99)**	**1.17** **(1.04, 1.31)**	**1.23** **(1.09, 1.39)**	1.00 (0.89, 1.13)	1.00 (0.89, 1.13)	0.96 (0.86, 1.08)	0.98 (0.88, 1.09)	**0.80** **(0.71, 0.89)**	**0.83** **(0.74, 0.92)**	1.07 (0.95, 1.21)	1.01 (0.89, 1.14)

^1^ The values shown are odds ratio (OR) and 95% confidence intervals (CI). Values in bold are significantly different at a level of *p* < 0.05. T1, T2, and T3 indicate the tertiles of dietary pattern scores.

## Data Availability

Publicly available datasets were analyzed in this study. This data can be found here: http://enutrition.fnri.dost.gov.ph/site/home.php (accessed on 14 February 2020).

## Supplementary Materials

The following supporting information can be downloaded at: https://www.mdpi.com/article/10.3390/nu14040886/s1, Figure S1: Scree plot showing variances of the factors extracted using factor analysis; Figure S2. Scree plot showing the percentage of explained variances of the dimensions or components extracted using principal component analysis; Table S1: Eigenvalues of the factors or components extracted using factor analysis; Table S2. Eigenvalues of the dimensions or components extracted using principal component analysis.
