# Supplementary material for: Dietary Patterns and Their Association with Sociodemographic and Lifestyle Factors in Filipino Adults
Source: Nutrients. 2022 Feb 19;14(4):886. doi: 10.3390/nu14040886 (PMC8876325; doi:10.3390/nu14040886)
Supplement: Supplementary file 1 [file nutrients-14-00886-s001.zip › nutrients-1578235-supplementary.pdf]

SUPPLEMENTARY MATERIAL

Dietary pattern extraction using factor analysis

The dietary patterns were derived through factor analysis in R software using the psych package (library(psych)). For the factoring method, we used the principal axis factoring. The varimax rotation was also applied. In determining the number of factors to extract, eigenvalues and scree plot were employed, as these are the two most common methods. The eigenvalues and scree plot of the factor solution are summarized in **Table S1** and **Figure S1**, respectively.

Sensitivity analysis

Sensitivity analysis was conducted to assess the stability of the six-factor solution generated from factor analysis. Principal component analysis (PCA) was carried out utilizing the actual data of the entire or whole sample. The ade4 package and dudi.pca function in R software were used. **Table S2** presents the eigenvalues for the 18 dimensions or components, while **Figure S2** displays the scree plot. Results of the PCA suggest a similar number of components (n = 6).

Table S1. Eigenvalues of the factors or components extracted using factor analysis.

Factor or Components	Eigenvalues
1	1.835
2	1.378
3	1.357
4	1.209
5	1.102
6	1.059
7	0.993
8	0.989
9	0.967
10	0.936
11	0.904
12	0.889
13	0.860
14	0.802
15	0.797
16	0.712
17	0.683
18	0.529

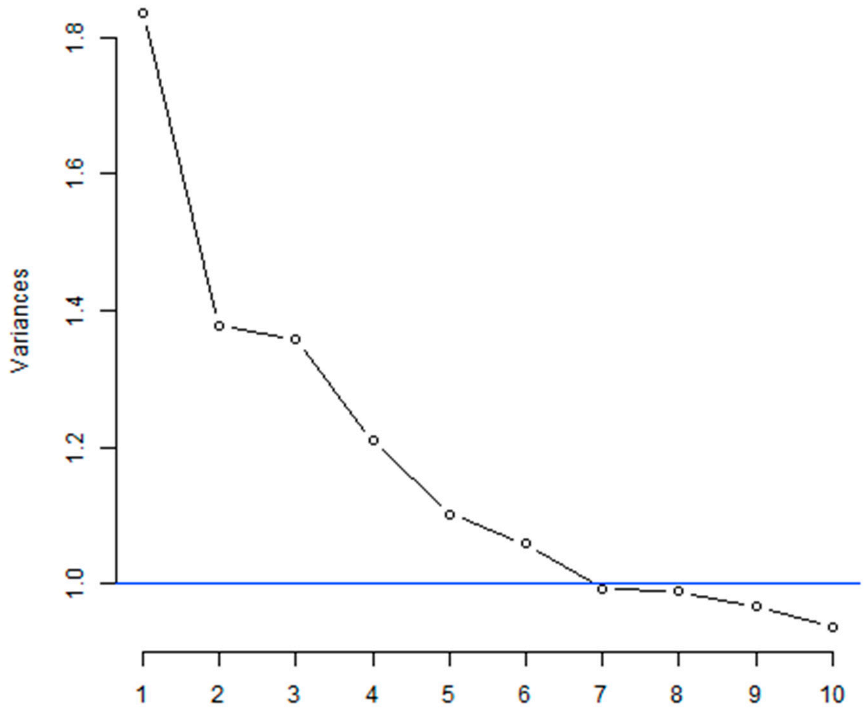

Figure S1. Scree plot showing variances of the factors extracted using factor analysis.

Table S2. Eigenvalues of the dimensions or components extracted using principal component analysis.

Dimensions	Eigenvalues
1	1.835
2	1.378
3	1.357
4	1.209
5	1.102
6	1.059
7	0.993
8	0.989
9	0.967
10	0.936
11	0.904
12	0.889
13	0.860
14	0.802
15	0.797
16	0.712
17	0.683
18	0.529

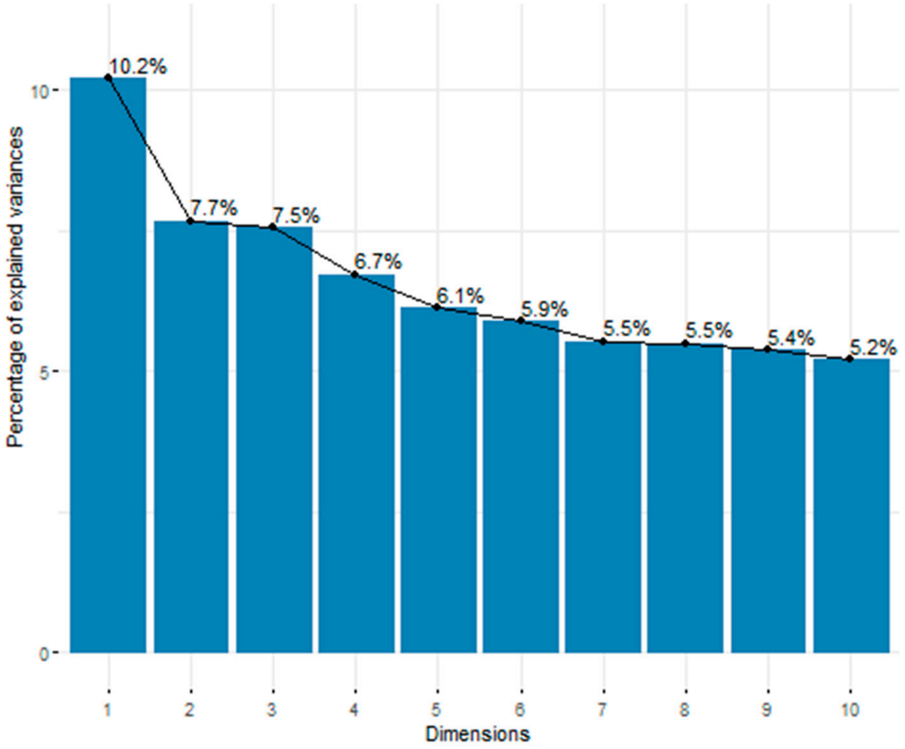

Figure S2. Scree plot showing the percentage of explained variances of the dimensions or components extracted using principal component analysis.
